# Antisecretory factor in severe traumatic brain injury (AFISTBI): protocol for an exploratory randomized placebo-controlled trial

**DOI:** 10.1186/s13063-025-08760-7

**Published:** 2025-02-07

**Authors:** Linus Réen, David Cederberg, Niklas Marklund, Edward Visse, Peter Siesjö

**Affiliations:** 1 Department of Clinical Sciences Lund, Neurosurgery, Lund University, Lund, Sweden; 2 Department of Neurosurgery, Skane University Hospital, Lund, Sweden

## Abstract

**Background:**

Despite recent advances in neuroimaging and neurocritical care, severe traumatic brain injury (TBI) is still a major cause of severe disability and mortality, with increasing incidence worldwide. Antisecretory factor (AF), commercially available as Salovum®, has been shown to lower intracranial pressure (ICP) in experimental models of, e.g., TBI and herpes encephalitis. The aim of this study is to assess the effect of antisecretory factors in adult patients with severe TBI on ICP and inflammatory mediators in extracellular fluid and plasma.

**Methods/design:**

This is a single-center, randomized, placebo-controlled clinical phase 2 trial, investigating the clinical superiority of Salovum® given as a food supplement during 5 days to adults with severe TBI (Glasgow Coma Scale (GCS) < 9), admitted to the neurocritical intensive care unit (NICU) at Skane university hospital, Lund, Sweden. All patients with GCS < 9 and clinical indication for insertion of ICP-monitor and microdialysis catheter will be screened for inclusion and assigned to either the treatment group (*n* = 10) or placebo group (*n* = 10). In both groups, the primary outcome will be ICP (mean values and change from baseline during intervention), registered from high-frequency data monitoring for 5 days. Secondary outcomes will be inflammatory mediators in plasma and intracerebral microdialysis perfusate days 1, 3, and 5 during trial treatment.

**Trial registration:**

ClinicalTrials.gov NCT04117672. Registered on September 17, 2017. Protocol version 6 from October 24, 2023.

**Supplementary Information:**

The online version contains supplementary material available at 10.1186/s13063-025-08760-7.

## Introduction

Despite recent advances in neuroimaging and neurocritical care, severe traumatic brain injury (TBI), frequently referred to as a silent epidemic, remains a major cause of severe disability and mortality with increasing incidence worldwide [1–6]. Cerebral edema is an important secondary injury mechanism, which accounts for an essential part of the morbidity and mortality in severe TBI [7]. The complex pathophysiological mechanisms causing the development and brain edema in severe TBI are poorly understood [2, 8–10]. However, the development of edema may cause raised intracranial pressure (ICP) that is considered critical with impact on both perfusion and diffusion in the brain [8, 9], resulting in deleterious effects on brain function [11].

Anti-secretory factor (AF) is an endogenous anti-inflammatory protein [12] that has shown clinical effects on Ménière’s disease and childhood diarrhea [13–15]. The exact mechanisms underlying the effects of AF are yet to be discovered, however, modulation of ion pumps, proteasomes, complement factors, and myeloid cells have been proposed [16]. In experimental models of TBI and herpes encephalitis, AF reduces ICP and improves outcomes [17, 18]. The AF protein is cleaved into several active peptides, one of which has been synthesized within a 16 amino acid peptide (AF-16) that has been used in animal experimental studies [17]. Salovum® (19) is a product based on the egg yolk powder B221® and contains high levels of AF. Salovum® is classified as a food for special medical purposes (FSMP) by the European Food Safety Agency. Salovum® has been given to hundreds of patients in clinical trials without any reports of adverse events [14, 15, 19, 20]. Egg yolk allergy is a contraindication, but no cases of triggered allergy have been reported. Beneficial effects of Salovum® in patients with severe TBI have been reported in small case series [21, 22] and a recently completed randomized trial has studied the effect of Salovum® on 30-day mortality in patients with severe TBI [23]. However, sampling ICP values once per hour is a crude measurement, and none of these studies have recorded ICP using frequency data sampling and analysis of inflammatory mediators. If this trial can demonstrate effect of Salovum® on ICP, a new and safe therapeutic option for ICP management in clinical practice would be available.

## Methods/design

### Trial design

This is a single-center, investigator-sponsored, phase 2, randomized, placebo-controlled, parallel-arm trial to assess the superiority of AF given as Salovum® in patients with severe TBI. The allocation ratio is 1:1. Recruitment commenced in March 2020.

### Trial population and eligibility

A total of 20 patients with severe TBI will be enrolled at a single study site, Skane University Hospital, Lund Sweden. Patients with a Glasgow Coma Scale (GCS) score < 9 and clinical indication for insertion of ICP monitor, intracerebral oxygen pressure monitor (Licox®), and microdialysis catheter will be screened for inclusion.

### Inclusion criteria

The inclusion criteria are as follows: patients with severe TBI, i.e., GCS < 9 at admission to NICU or within 24 h of injury; planned for neurointensive care including ICP-monitoring, microdialysis, and intracerebral oxygen pressure monitoring; aged between 10 and 70 years; pathological findings on initial CT scan.

### Exclusion criteria

The exclusion criteria are as follows: known egg yolk allergy; unilateral or bilateral fixed and dilated pupil(s) after initial surgical intervention; non-fulfillment of inclusion criteria after screening.

### Management of traumatic brain injury

The study site will treat all study patients according to the hospital standard care that may include assisted ventilation, use of invasive ICP monitors, head elevation, hyperventilation, barbiturate coma, drainage of cerebrospinal fluid (CSF), and surgical measures to lower ICP, including decompressive craniectomy.

### Study participants

Study participants will be composed of 20 patients with severe TBI (GCS < 9) who are planned for neurointensive care, including ICP monitoring, microdialysis, and intracerebral oxygen pressure monitoring. When a patient with severe traumatic brain injury is admitted to the neurointensive care unit, the neurosurgeon on call or study investigator will evaluate the patient, provide next of kin with informed consultation, and use the screening protocol for inclusion. For patients < 18 years, signed informed consent by next of kin is required for inclusion.

### Ethics and protocol

Ethical approval has been granted by the Swedish ethical review authority (2019/1). The study will comply with the ethical principles as set down in the Declaration of Helsinki and will be conducted in accordance with good clinical practice as defined by the International Conference on Harmonization (ICH). The trial is registered with ClinicalTrials.gov, NCT04117672; any amendments to the protocol will be published on ClinicalTrials.gov.

### Randomization

Patients in the trial are allocated to treatment with Salovum® or placebo egg yolk powder at a ratio of 1:1. The allocation sequence is generated by the principal investigator. Permuted block randomization, with blocks of 4–6, will be used and was compiled with R. Each patient is assigned a sequentially numbered study binder. The study number written in the binder corresponds to a sealed and opaque box with the same number, containing the study substance, either active (Salovum®) or placebo (normal egg yolk powder). Study substance and placebo appear identical with respect to packaging, color, texture, and smell. Investigators, caregivers, and patients are blinded during the entire trial. If any patient needs unblinding for medical reasons, an emergency unblinding list exists in digital form.

### Trial interventions

#### Active therapy

The active therapy is Salovum®, an egg yolk powder enriched for AF, which is manufactured from freeze-dried egg yolk (Lantmännen Functional Foods AB, Stockholm, Sweden).

#### Placebo therapy

A placebo powder, containing low amounts of antisecretory factor, made from freeze-dried egg yolk and identical in taste, texture, smell, and color to Salovum®, will be used. The placebo powder is manufactured and supplied by Lantmännen Functional Foods AB.

#### Dosage

Salovum® or placebo egg powder will be administrated for 5 days. The trial substance is administered according to 1 g per patient kg body weight per 24 h, divided into 6 dosages, administered every 4 h. The study site is equipped with digital scales for weighing the trial substance before administration.

#### Dispensing

Both the Salovum® and placebo substance are packaged in identical bags by Lantmännen Functional Foods AB. After opening of a bag, each containing 20 g, a weighed aliquot depending on patient weight will be mixed with 50–100 ml tap water in a glass container. Electrical milk frothers are used for mixing. The mixture will then be aspirated into a syringe and administered to the patient via the nasogastric tubing, used for enteral nutrition.

#### Protocol adherence

Protocol adherence will be encouraged via monitoring and daily inspections by investigators and/or research nurses. Any important protocol modifications will be promptly communicated to all study investigators and research nurses.

#### Study endpoints

The primary endpoint is the effect of AF, given as a dietary supplement in the form of Salovum®, compared with placebo on ICP [24]. The secondary endpoint is the effect on inflammatory mediators [25] in patients with severe TBI. Exploratory endpoints include intracerebral oxygen pressure (PbtO_2_) [26], cerebral perfusion pressure (CPP), mortality at 30 days, therapy intensity level (TIL, see page 9) [27, 28], brain metabolites [29, 30], ICP change from baseline, ICP under the curve and outcome according to the extended Glasgow Outcome Scale (eGOS) at 6 months. Binary outcome variables (i.e., 30-day mortality) will be analyzed using the chi-square test. For continuous data, the Mann–Whitney *U* test will be used. All computations will be run with the free statistical software R (https://www.r-project.org).

#### Data collection of outcome parameters and predefined covariates

**Fig. 1:**
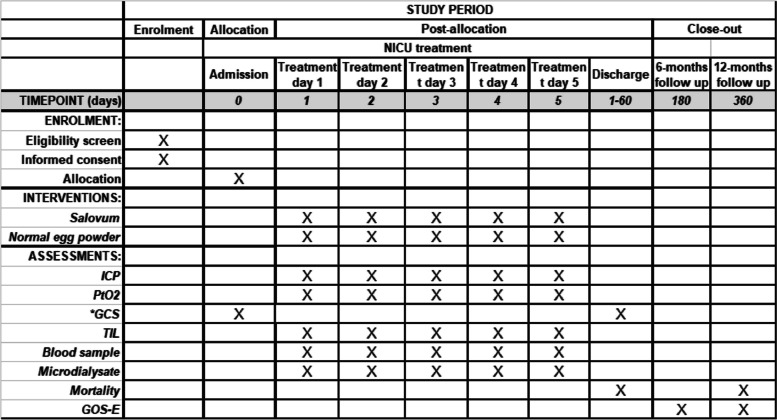
SPIRIT figure. * Intubated patients are evaluated using only the sum of eye opening and motor responses (GCS-T)

Basic parameters (GCS, GCS-T, age, gender, medical history, diagnosis) are collected from electronic medical charts (Melior) and the monitoring system (IntelliSpace Critical Care and Anesthesia [ICCA] system; Philips) at the NICU. High-frequency data collection of physiological parameters (ICP, CPP, PbtO_2_, pulse, blood pressure, PO_2_, temperature) are sampled with a Moberg® CNS monitor. Brain metabolites (Lactate/pyruvate ratio, glucose, glutamate, glycerol) are analyzed using ISCUS flex (M Dialysis AB). All data from the Moberg CNS monitor and other electronic sources will be transferred to an electronic CRF (Redcap). TIL will be scored every 24 h. TIL will be recorded in a paper CRF and then transferred to the electronic CRF continuously., Plasma samples will be collected before the initial dose of the trial substance, thereafter twice daily for 5 days, and after the last dose of the trial substance. After centrifugation, aliquoted samples will be stored in a − 80 °C freezer. The plasma will be analyzed for markers of brain damage and cytokines/chemokines. Similarly, microdialysis perfusate fluid will be stored in a − 80 °C freezer and analyzed for cytokines/chemokines and markers for brain damage (see Tables 1 and 2). Age, gender, GCS, GCS-T, and trauma mechanism at inclusion, may be used as covariates in exploratory regression analysis. Medical charts are used to track 30-day mortality. Patients or next of kin are followed up by visits and/or telephone interviews at the outpatient clinic in order to assess eGOS > 6 months. All data is stored in a specific password-protected eCRF, only accessible to investigators (Fig. 1).

**Table 1:** Primary and secondary outcome measures

Outcome measure	Measure description	Time frame	Objective
ICP mean	Measured by an intracranial pressure sensor	During intervention, 5 days	Primary
ICP area under curve	Measured by an intracranial pressure sensor	During intervention, 5 days	Primary
Inflammatory cytokine secretion	Interleukin-6 (Il-6), interleukin-8 (IL-8), Interferon-gamma inducible protein (IP10) and monocyte chemotactic protein (MCP-1) assessed from microdialysate and plasma by multiplex analysis	During intervention, 5 days	Secondary

**Table 2:** Exploratory outcome measures

Outcome measure	Measure description	Time frame
Mortality	Mortality due to TBI	At 30 days and 12 months
Morbidity	Assessed by Glasgow Outcome Scale Extended (GOSE)	At 6 and 12 months
Intracerebral oxygen partial pressure	Measured by an intracranial oxygen sensor	Change from baseline during intervention
Intracerebral oxygen partial pressure	Measured by an intracranial oxygen sensor	During intervention, 5 days
Treatment intensity level	Treatment intensity level (TIL) scale. Minimum 0 (no intervention to control intracranial pressure (ICP)), maximum 38 points (maximum efforts to control ICP)	Change from baseline during intervention
Treatment intensity level	Treatment intensity level (TIL) scale. Minimum 0 (no intervention to control intracranial pressure (ICP)), maximum 38 points (maximum efforts to control ICP)	During intervention, 5 days
Rate of cerebral metabolism	Lactate/pyruvate ratio assessed by online microdialysis	Change from baseline during intervention
Rate of cerebral metabolism	Lactate/pyruvate ratio assessed by online microdialysis	During intervention, 5 days
Concentration of brain damage markers	Glial fibrillary acidic protein (GFAP), neurofilament light protein (NFL), tubulin associated unit (TAU), and ubiquitin C-terminal hydrolase L1 (UCH-L1)	Change from baseline during intervention
Concentration of brain damage markers	Glial fibrillary acidic protein (GFAP), neurofilament light protein (NFL), tubulin associated unit (TAU), and ubiquitin C-terminal hydrolase L1 (UCH-L1)	During intervention, 5 days
ICP mean	Measured by an intracranial pressure sensor	Change from baseline during intervention
ICP area under curve	Measured by an intracranial pressure sensor	Change from baseline during intervention
Inflammatory cytokine secretion	Interleukin-6 (Il-6), interleukin-8 (IL-8), interferon-gamma inducible protein (IP10) and monocyte chemotactic protein (MCP-1) assessed from microdialysate and plasma by multiplex analysis	Change from baseline during intervention

### Definition of outcome measures

#### Intracranial pressure (ICP)

ICP is measured with an inserted catheter for recording both intracerebral and, if possible, intraventricular pressure. Intracranial pressure measurement is a clinical routine in the treatment of severe head injury.

#### Cerebral tissue oxygen pressure (PbtO2)

PbtO_2_ is measured with inlaid probe (Licox, Integra) and is a clinical routine for a severe head injury.

#### RLS (Reaction Level Scale)

RLS is a certified scale for assessing the depth of unconsciousness and is a clinical routine for severe head injury in Nordic countries. The RLS scale goes from awake [1] to brain death/reactionless [8]. RLS can be translated as GCS.

#### GCS (Glasgow Coma Scale)

GCS is a certified scale for assessing the depth of unconsciousness in severe head injury internationally. GCS can be translated as RLS.

#### GCS-T

GCS-T is a modified GCS for intubated patients, evaluating only eye opening and motor responses.

#### Microdialysis of the brain

Microdialysis is done with thin, membrane-equipped catheters which are introduced into the brain tissue. Metabolic factors can be measured online and brain injury markers, inflammatory factors, etc. can be analyzed afterwards after the samples are frozen. Microdialysis is a clinical routine in severe head injuries. In this study, the lactate/pyruvate ratio and the glucose content are mainly analyzed.

#### Glasgow Outcome Scale - Extended (GOSE)

GOSE is a validated scale for estimating capacity and function in the follow-up of patients after head injury. The scale goes from 1 (dead) to restored 8 (good outcome).

#### Therapy Intensity Level (TIL)

TIL is a validated scale for estimating the measures taken to maintain normal intracranial pressure. The scale goes from no actions (0p) to full actions (38p). Low values indicate good control of ICP, high values indicate poor control of ICP.

#### Inflammatory mediators

Inflammatory cytokines are analyzed from microdialysates and blood using single and multiplex techniques; Mesoscale (Mesoscale Discovery) and O-link (O-link proteomics).

#### Brain injury markers

Brain injury markers, such as Nf-L, TAU, GFAP, and UCH-L1, are analyzed in blood and microdialysates using single ELISA or single/multiplex technique as described above.

#### Statistical power of proposed endpoints

This is an exploratory trial with no previous data to calculate power.

#### Statistical analysis

Binary outcome data (e.g., mortality) will be analyzed with the chi-square test. Continuous data will be analyzed using the Mann–Whitney *U* and Wilcoxon tests. Both per-protocol and intent-to-treat analyses will be performed. All statistical calculations will be conducted in R Studio. Please see Additional files 1 and 3 for detailed information.

#### Handling of missing data

With the exception of TIL, the data collected in this trial is limited to what is normally measured and registered during standard care at the NICU. Therefore, the amount of missing data is expected to be low. Data missing at random will be handled using the last observation carried forward (LOCF). Data not missing at random will be analyzed using mean substitution. If microdialysis is not initiated or fails after insertion, the patient will be given the trial substance or placebo as scheduled, but additional patients will be added in order to achieve 20 patients monitored with microdialysis data for a minimum of 3 days.

#### Strategies to achieve adequate participant enrolment

Skane University Hospital, Lund, is a tertiary unit with full neurosurgical capacity that serves approximately 2 million people and treats approximately 15 patients with severe TBI yearly. The use of ICP monitoring, microdialysis, and intracerebral oxygen pressure monitoring is standard clinical practice for the treatment of severe TBI.

#### Handling of protocol deviations and protocol violations

We defined 4 types of possible protocol deviations/violations in this trial: faulty enrolment of patients (enrolment of ineligible patients), faulty randomization (if patients are given the wrong randomization number), faulty intervention (if patients are given wrong intervention and/or do not adhere to the protocol), and faulty data collection. The primary investigator is responsible for enrolment and randomization of patients. TIL is noted daily by investigators. Daily visits are made by the investigator and/or research nurse to ensure correct administration of trial substance, sampling of blood/perfusate, and data collection. Any protocol violation/deviation will be disclosed at the end of the trial as described in previous paragraphs.

#### Adverse events

As Salovum® is commercially available in Swedish pharmacies and has been available for human use for many years without any reported toxicity, it is not expected to cause any adverse events. Allergy to egg yolk protein (Gal 5; alpha-livetin) has an estimated low incidence in adults (< 0.1%), and anaphylactic reactions have not been reported. However, special care will be taken to ensure that no vital parameters are changed for the worse in conjunction with the administration of the active substance or placebo and at the time for each dose administration, i.e., every 4 h. The physician responsible for the patient will assess if any adverse or serious adverse events have occurred during the course of each day. The placebo powder is identical to Salovum®, except for significantly lower levels of AF and AF-derived peptides, and is therefore expected to have a similar safety profile.

Adverse events will be classified according to CTCAE v.5.0. Allergic adverse events will be classified as follows; adverse events: skin rash and hives; serious adverse events: serious anaphylactic reaction with hypotension and bronchospasm requiring intervention with corticosteroids and/or vasopressors.

#### Data and trial monitoring and interim analysis

No interim analysis will be performed. The trial will be monitored by the Centre for Clinical Trials-Forum South, Skane University Hospital.

## Discussion

The mechanisms and pathological processes behind the deleterious effects on the severely damaged human brain are yet to be understood [8–10, 31]. Current therapies (e.g., sedation, hyperventilation, hyperosmolar treatment, and decompressive craniectomy) are non-targeted and have serious side effects [10, 32]. Although the knowledge of causal mechanisms underlying secondary injury in severe TBI is limited, molecular candidates targeting the underlying pathophysiology of cerebral edema have been proposed [9, 10, 33]. Nevertheless, no specific drug has been shown to decrease ICP or improve outcomes in severe TBI [31, 34]. The effect of AF on 30-day mortality has recently been studied by Cederberg et al. (submitted manuscript) [23] however, a more in-depth analysis of ICP has never been conducted. This warrants dedicated studies on AF’s effect on ICP and cerebral edema in severe TBI.

PbtO_2_ has been extensively used in the monitoring of severe TBI with the assumption that hypoxia may be an independent deleterious factor during periods of controlled ICP [26]. Tumor hypoxia has been linked to raised interstitial fluid pressure, which has been shown to be regulated by AF-16 [35]. Hence measurement of PbtO_2_ is relevant in the present trial to reveal changes unrelated to altered ICP. TBI induces a potent intracerebral inflammatory response that is mostly confined to the brain [25]. Whether this response partially triggers the induced cerebral edema is plausible but not yet proven. Factors such as VEGF, IL-8, and IL-6 which are upregulated after TBI also have a profound effect on vascular integrity and fluid extravasation(36, 37). The anti-inflammatory and antisecretory properties of AF have been known since the 1980s [12]; however, its role in the treatment of cerebral edema remains unclear. Furthermore, the ability to reduce ICP has only been shown in animal studies and small pilot studies of severe TBI patients (17, 18, 21, 22). Therefore, it is of utmost interest to assay inflammatory cytokines after treatment with Salovum®.

This is, to our knowledge, the first randomized study to thoroughly explore the anti-inflammatory and ICP-reducing effects of AF using high-frequency data sampling of ICP and PbtO_2,_ and continuous sampling of microdialysate during the first critical days after severe TBI. This study aims to reveal the effects of AF on neuroinflammation in the severely injured brain, and if previous effects on ICP can be confirmed, it would be highly supportive of further use of AF in severe TBI.

## Trial status

The protocol is version 6, dated September 28, 2023. This trial is active and has been recruiting since March 11, 2020. Recruitment is estimated to be completed on December 30, 2024.

## Supplementary Information

Additional file 1:

Additional file 2: Informed consent

Additional file 3: SAP AFTBI Trials

## Data Availability

The full protocol is available as Additional file 1. The full anonymized data set will be available to the public upon reasonable request.
