# Supplementary material for: Antisecretory factor in severe traumatic brain injury (AFISTBI): protocol for an exploratory randomized placebo-controlled trial
Source: Trials. 2025 Feb 7;26:43. doi: 10.1186/s13063-025-08760-7 (PMC11804074; doi:10.1186/s13063-025-08760-7)
Supplement: Supplementary file 1 — Additional file 1: [file 13063_2025_8760_MOESM1_ESM.docx]

# Treatment of severe brain injury with antisecretory factor

## Research protocol

### Scientific questions

#### Primary aims

1. To investigate whether treatment with Salovum^®^, an egg powder enriched with antisecretory factor, reduces intracranial pressure in severe traumatic brain injury (TBI).

#### Secondary aims

1. To investigate whether treatment with Salovum^®^ reduces inflammatory cytokines in extracellular fluid, obtained using microdialysis and plasma.

#### Exploratory aims

1. To investigate whether the administration of Salovum reduces mortality after 30 days and 6 months.
2. To investigate whether administration of Salovum reduces morbidity according to GOSE at 6 and 12 months.
3. To investigate whether treatment with Salovum^®^, an egg powder enriched with antisecretory factor, affects intracerebral oxygen pressure and in severe traumatic brain injury.
4. To investigate whether treatment with Salovum^®^ reduces treatment intensity level (TIL) in severe traumatic brain injury.
5. To investigate whether treatment with Salovum^®^ affects brain metabolites (lactate/pyruvate ratio and glucose) and brain injury markers in microdialysate and plasma.

## Background

Traumatic brain injury (TBI) is an essential part of severe morbidity and death worldwide and the cost of TBI has been estimated at 0.5% of world GDP. The mortality rate in severe TBI varies between 10 and 70% and at best 20% of patients regain the same functions and abilities as before the injury.

Brain swelling/cerebral edema occurs both in trauma and other diseases of the brain as stroke and meningitis. The cerebral edema leads to an elevated intracranial pressure (ICP) with an impact on both cerebral blood flow (perfusion) and diffusion in the brain. Untreated or uncontrolled, cerebral edema can lead to herniation and death. The mechanisms behind the onset of cerebral edema/swelling are not fully known, but excitotoxic (via massive release of naturally occurring stimulating transmitter substances such as glutamate) as well as inflammatory and ischemic causes have been suggested. The development of the edema is believed to occur gradually from an initial intracellular edema, followed by an ionic edema, a vasogenic edema, and a hemorrhagic edema/progressive secondary hemorrhage and a hemorrhagic edema. Current treatment can only partially limit secondary consequences of the cerebral edema. Treatment with removal of injured brain tissue and hemorrhages as well as removal of the skull bone (a decompressive craniectomy) often needs to be performed but involves an increased risk of per- and postoperative complications.

AF - antisecretory factor- is a 41kDa large endogenous protein that was first discovered due to its ability to inhibit experimental diarrhea. Endogenous AF activity increases after exposure to bacterial toxins and increased AF activity along with an immune reaction may be part of the normal defense against the secretory and inflammatory component of diarrheal disease. The AF protein is cleaved into several active peptides, one of these has been synthesized as a 16 amino acid long peptide (AF-16) used in experimental studies as shown below. Salovum® is a product based on the egg yolk powder B221® and contains high levels of AF. Salovum® is classified as a "medical food supplement" by the EU and can be purchased without a prescription at pharmacies in Sweden.

In addition to the effect on secretory diarrhea, AF has shown efficacy in Meniere´s disease, mastitis and meningitis. In TBI models, AF has been shown to be effective at reducing elevated ICP to virtually normal values and also improved functional outcome. Similarly, AF has been shown to reduce ICP and increase survival in an experimental model of herpes encephalitis. Our preliminary results show that treatment with antisecretory factor in patients with severe TBI reduces intracranial pressure and improves treatment outcome. A randomized, prospective, phase 2-3 trial on the effect of AF on 30-day mortality in patients with severe TBI has been completed at Tygerberg University Hospital, Cape Town, South Africa (submitted manuscript). Since Salovum^®^ is not classified as a medicinal product, approval from the medicinal agencies is not requested.

Antisecretory factor is an endogenous protein and no antibody formation has been detected during administration to humans. Despite the fact that Salovum^®^ has been given to hundreds of patients, no side effects have been recorded. Yolk allergy is a contraindication, but no cases of triggered allergy have been reported.

How antisecretory factor reduces brain swelling is not understood. It has been suggested that antisecretory factor peptides inhibit specific ion pumps, that they stabilize/suppress a certain type of white blood cell, macrophages, and that they affect circulating complexes of complement factors and proteasomes. Experiments on isolated membranes and cells show that AF has no direct effect on these arguing for other mechanisms of action. Pharmacokinetic factors also suggest a rapid secondary effect directed against factors that create the brain edema.

## Study plan and procedures

Study type

Prospective, randomized, phase 2 study. Patients, caregivers and investigators are blinded during the entire trial.

Illness

Severe traumatic brain injury, defined as loss of consciousness, which according to the Glasgow Coma Scale (GCS) is estimated as ≤ 8 at admission.

Patients

10+10 =20 patients. Ages 10-70 can be included in the study.

Interventions

Oral administration of Salovum® or placebo egg powder. 1 g/kg daily for 5 days

Study substance.

Salovum® is given orally 6 times daily, at a dose of 1g/kg/day. Patients are administered Salovum® or placebo egg powder (identical packaging, color, consistency and smell) via naso-gastric probe (placed according to clinical routine) after mixing in at least 100 ml of water/dose.

### Centres

Neurointensive care unit (NIVA), Skane University Hospital (SUS).

### Inclusion criteria

1. Patients with severe traumatic brain injury, GCS<9 at admission and treated at NIVA. Patients under the age of 18 who are treated at NIVA are also eligible may be included.
2. Clinical indication for insertion of intracranial pressure gauge, catheter for measuring intracerebral oxygen pressure and microdialysis catheter.
3. Consultation with a relative or consent from a guardian.

### Exclusion criteria

1. Known egg yolk allergy
2. Unilateral or bilateral light rigid and dilated pupil after initial neurosurgical operative intervention
3. Age > 70 years
4. Age < 10 years

### Randomization.

Block randomization for Salovum or normal egg powder. Randomization binders numbered from 1-20.

### Patient follow-up and definition of outcome measures

Patients included in the study will be followed up according to Table 1.

*Table 1. CRF*

| CRF | **Day 1-5** | **Discharge NICU*** | **30 days** | **6 months** | **12months** |
| --- | --- | --- | --- | --- | --- |
| ICP | x |  |  |  |  |
| PbtO2 | x |  |  |  |  |
| RLS+GCS |  | x |  |  |  |
| TIL | x |  |  |  |  |
| Blood sample | x |  |  | x |  |
| Microdialysate | x |  |  |  |  |
| Mortality |  |  | x |  | x |
| GOS-E |  |  |  | x | x |

ICP – Intracranial pressure, PbtO_2_ – Cerebral tissue oxygen pressure, RLS – Reaction level scale, GCS – Glasgow coma scale, TIL – Therapy intensity level, GOS-E – Glasgow outcome scale extended

_* Intubated patients are evaluated using only the sum of eye opening and motor responses (GCS-T) ‘_

### Data collection and data storage

Data from online monitoring (ICP, Pt0 2, pulse, blood pressure, oxygen saturation, temperature, PC0_2_, microdialysis metabolites will be stored via specific equipment (CNS Monitor, Moberg ICU solutions, Moberg) and dedicated server. The server is only accessible via a specific password and network address and only accessible to the doctor responsible for studies. Other data will be retrieved from existing documentation portals (Melior, ICCA) or registered by study-responsible physicians at NIVA, ward or return visits. All data will be collected in a database (FileMaker Pro) which is both password and URL protected as described above. Data will only be stored in encoded condition. All data will be processed according to GDPR.

## Statistics

Binary outcome data (mortality) will be analyzed using the chi-square test. Continuous outcome data (other data) will be analyzed with the Mann-Whitney U test.

All statistical analyses will be performed with R-project (<https://www.r-project.org)>.

## Final analysis

Final analysis will be done after the last patient 30 days of follow-up.

## Significance

There is no effective treatment for cerebral oedema in severe head injury. The study aims to investigate the mechanisms underlying the effect of antisecretory conditions in the treatment of severe head injury.

## Previous experience of methods, procedures (own and others) especially with regard to risks and possible complications.

5 patients with severe head injury have been treated with Salovum^®^ via nasogastric probe. In three of the patients, a rapid decrease in intracranial pressure was seen within 24 hours and advanced treatment such as decompressive craniectomy and pentothal therapy could be avoided. In two of the patients, a clear effect was seen in periods without ventricular retention (gastroparesis) and poorer efficacy in ventricular retention. A prospective randomized study on severe traumatic head injury with Salovum^®^ has recently been completed in Tygerberg University Hospital, Cape Town, RSA. In experimental brain tumor models, intratumoral AF-16 treatment increases survival and in combination with intratumoral chemotherapy, > survives 90%. The ethical state of a prospective study in 100 patients with high-grade primary brain tumors, glioblastoma, has recently been approved and the study is expected to begin in spring 2019.

## Security/personnel

No security issues for staff can be identified or predicted.

## Ethical considerations

Antisecretory factor has shown promising results experimentally and tentatively in clinical treatment. No side effects of treatment with Salovum^®^ have been reported. Since unconsciousness > 6 hours is part of the inclusion criteria, all patients will be incapacitated. We will therefore consult with relatives or ask guardians (children<18 years) after written and oral information. The outcome of consultations with relatives will be noted in the medical record. When the patient becomes capable of making decisions, they will be asked for continued participation in the study. As in all studies in serious, fatal diseases, there is an ethical consideration to give a hope for the effect of the treatment. The oral and written information strongly states that there are no guarantees of clinical efficacy and that neither the investigator nor the patient will know what treatment has been given before the interim analysis.

## Conflict of interest

All processing of data will be performed in accordance with GDPR. There will be no patient remuneration for participation in this study. None of the investigators or sponsors will benefit financially from the conduct of this trial. All analyses, interpretations and publications of data will be performed independently by the investigators. The investigators have no other competing interests or conflicts of interest to declare. Funding to cover costs for screening, inclusion, treatment and follow-up will be supplied by research funds from the Department of Neurosurgery, Skane University Hospital, Sweden.

## References

1. Kaya I, Johansson E, Lange S, Malmberg P. Clinical Nutrition Experimental. Elsevier Ltd; 2017 Apr 1;12:27–36.
2. Ilkhanizadeh S, Sabelström H, Miroshnikova YA, Frantz A, Zhu W, Idilli A, et al. Antisecretory Factor-mediated Inhibition of Cell Volume Dynamics Produces Anti-tumor Activity in Glioblastoma. Molecular Cancer Research. 2018 Feb 5.
3. Hansson H-A, Al-Olama M, Jennische E, Gatzinsky K, Lange S. The peptide AF-16 and the AF protein counteract intracranial hypertension. Acta Neurochir Suppl. 2012;114:377–82.
4. Clausen F, Hansson H-A, Raud J, Marklund N. Intranasal Administration of the Antisecretory Peptide AF-16 Reduces Edema and Improves Cognitive Function Following Diffuse Traumatic Brain Injury in the Rat. Front Neurol. 2017;8:39.
5. Al-Olama M, Wallgren A, Andersson B, Gatzinsky K, Hultborn R, Karlsson-Parra A, et al. The peptide AF-16 decreases high interstitial fluid pressure in solid tumors. Acta Oncol. 2011 Oct;50(7):1098–104.
6. Al-Olama M, Lange S, Lönnroth I, Gatzinsky K, Jennische E. Uptake of the antisecretory factor peptide AF-16 in rat blood and cerebrospinal fluid and effects on elevated intracranial pressure. Acta neurochirurgica. 2014 Sep 24.
