# Supplementary material for: Antisecretory factor in severe traumatic brain injury (AFISTBI): protocol for an exploratory randomized placebo-controlled trial
Source: Trials. 2025 Feb 7;26:43. doi: 10.1186/s13063-025-08760-7 (PMC11804074; doi:10.1186/s13063-025-08760-7)
Supplement: Supplementary file 2 — Additional file 2: Informed consent [file 13063_2025_8760_MOESM2_ESM.docx]

**Treatment of Severe Head Injury with Antisecretory Factor**

**Information for Relatives**

**Information for You as a Relative**
We would like to consult with you, the relative of a patient being treated at NIVA, SUS-Lund, for a severe head injury, regarding participation in a study where an antisecretory factor is administered as an addition to the treatment we have described above.
We are also asking for your consent to take 15 extra blood samples from your relative.
Finally, we are asking for permission to store your relative's blood samples in our biobanks and to analyze them in our laboratories in Lund or other laboratories in Sweden.

**Background and Purpose**
At the Neurosurgical Clinic in Lund, patients with severe head injuries are treated. In cases of severe head injury, the brain often swells, leading to increased pressure in the brain, which impairs blood circulation and the supply of oxygen and nutrients. If the pressure in the brain cannot be controlled, it can result in brain compression and loss of function. The treatment of patients with severe head injuries is guided by a protocol and includes sedation, monitoring of brain pressure, oxygen levels in the brain, and brain metabolism through inserted catheters. We also administer certain medications to reduce brain pressure. If these measures are insufficient to control brain pressure, we must operate on the patients to remove hemorrhages or a part of the skull bone. In some patients, we can control the brain pressure in this way, but unfortunately, in others, we cannot. Antisecretory factor is a naturally occurring protein in the body that increases during infections such as diarrheal diseases. Thousands of people have been treated with antisecretory factor in the form of an egg powder, Salovum, which contains large amounts of antisecretory factor. Salovum is not a drug but a dietary supplement, and no side effects have been reported with its use. Salovum can be purchased over the counter in pharmacies in Sweden and the EU. Research results from our own and other groups show that the antisecretory factor can reduce pressure in brain swelling following a head injury.

**How Will the Study Be Conducted?**
Since we do not know if the antisecretory factor will have any effect on your relative's condition, severe head injury, your relative will be randomly assigned to treatment with Salovum or regular egg powder with normal levels of the antisecretory factor. Neither you nor the treating physician will know which treatment is being given until after the last patient in the study has been treated. This may take over a year. Your relative will be given Salovum or regular egg powder six times daily for five days.
The egg powder is mixed with water and administered through the tube to the stomach that your relative already has.
If you know that your relative has had an allergic reaction to egg yolk (egg yolk allergy is very rare), unfortunately, your relative cannot participate in the study. The blood samples will be taken during the stay at NIVA and at a follow-up visit after 6 months. Otherwise, the same treatment as described above will be given as routine care.

**Significance for Your Relative**
Our aim is to determine whether the antisecretory factor can improve the treatment of severe head injury and, if so, how it works. If your relative receives the egg powder with high levels of antisecretory factor, Salovum, it could mean that your relative recovers better. However, we want to emphasize that there is currently no conclusive evidence that the addition of antisecretory factor to the routine treatment of severe head injury in humans has any effect.

**Handling of Your Relative's Blood Samples**
Participation in providing blood samples for the study is voluntary. Your relative's blood samples will be stored in an approved biobank in Lund. Biobanks are authorized collections of samples where patients' samples are labeled with a code. The key to the code is only accessible to the study's responsible physicians. The samples will be analyzed at laboratories in Lund, Uppsala, and other laboratories in Sweden. To avoid varying results, we aim to analyze as many samples as possible at the same time, but the analyses may need to be repeated at later dates. Before analysis, the samples will be stored for up to 5 years. If your relative's samples are relevant for analysis in a new study, you or your relative will be asked for consent separately. You can withdraw your consent at any time and have the samples destroyed if you wish. If you have further questions about the biobank, you can contact the study's responsible physicians.

**Handling of Data and Confidentiality**
Your relative's personal data will be recorded according to the rules in the EU's General Data Protection Regulation (GDPR). The data stored for the purposes of the study will be handled in a way that prevents unauthorized access. The data controller and responsible party under GDPR is Region Skåne. You have the right, free of charge, to access the information stored about your relative and to correct any inaccuracies. You also have the right to have data deleted or to limit the processing of your relative's data. You can contact one of the staff members at the Neurosurgical Clinics as listed below. The main person responsible for your data is the Data Protection Officer at Region Skåne, whom you can reach as follows:

**Data Protection Officer, Region Skåne, 291 89 Kristianstad**
**Phone:** 044-309 30 00
**Email:** region@skane.se

You also have the right to file a complaint with the Data Protection Authority if you are dissatisfied with how your personal data has been handled. You can reach the Data Protection Authority as follows:

**Data Protection Authority, Box 8114, 104 20 Stockholm**
**Phone:** 08-657 61 00
**Email:** datainspektionen@datainspektionen.se

**Voluntariness**
Your consent is voluntary. You can withdraw your consent at any time and discontinue your relative's participation in the study without it affecting your relative's other treatment. The results of the study may be presented at international conferences and published in scientific journals where your relative will not be identifiable as an individual. If you wish, you can be notified when publication has occurred.
If you have any questions, please feel free to contact one of the staff members at the Neurosurgical Clinic, Skåne University Hospital, as listed below.

**Responsible for the Study**
Peter Siesjö, Physician, Neurosurgical Clinic, Tel: 046-171274, Email: peter.siesjo@skane.se
David Cederberg
Linus Reen
Niklas Marklund
Isabella Holm

If you have no objections to your relative participating in the study, we kindly ask you to sign the attached consent form:

**Consent to Participate in the Study - Treatment of Severe Head Injury with Antisecretory Factor**

**Consent to Participate in the Study**
I have received verbal and written information about the study and have had the opportunity to ask questions. I will keep the written information.
☐ I consent to my relative participating in the study.
☐ I consent to my relative's data being processed as described in the participant information.
☐ I consent to my relative's samples being stored in a biobank as described in the participant information.

| Place and date | Signature |
| --- | --- |
|  |  |
