# Supplementary material for: Antisecretory factor in severe traumatic brain injury (AFISTBI): protocol for an exploratory randomized placebo-controlled trial
Source: Trials. 2025 Feb 7;26:43. doi: 10.1186/s13063-025-08760-7 (PMC11804074; doi:10.1186/s13063-025-08760-7)
Supplement: Supplementary file 3 — Additional file 3: SAP AFTBI Trials [file 13063_2025_8760_MOESM3_ESM.docx]

## Statistical analysis plan for clinical trial Antisecretory factor in severe traumatic brain injury (AFISTBI). Version 1.00

## Statistical methods

## All statistical calculations and sample size estimates were performed with the free statistical software R studio.

### *Baseline characteristics*.

Baseline data will be enlisted in a table without statistical comparison.

### *Study endpoints*

The primary endpoints is the effect of AF, given as a dietary supplement in the form of Salovum®, compared with placebo on ICP in adult patients with severe TBI. Comparisons will be made using Mann Whitney U test for comparisons between active and placebo arms.

Secondary endpoints are difference in secretion of inflammatory cytokines and will be computed as for primary outcomes. Significance will addressed per cytokine; IL-6, IL-8, IP-10, MCP-1, MCP-4, MIP1a, MIP1b, MIP3a, Eutaxon, Eotaxin3, MDC, TARC, Il-11, IL-16, VEGF-A. For comparisons between the groups, Mann-Whitney U test will be used.

For the exploratory endpoints chi-square test will be used for overall survival and Mann Whitney U and Wilcoxon signed rank tests for morbidity, TIL, intracerebral oxygen partial pressure and cerebral metabolism.

### *Handling of missing data*

With the exception of 30-day mortality, the data collected in this trial is limited to data that is normally measured and registered during standard care at the NICU. Therefore, the amount of missing data is expected to be low. Data missing at random will be handled using last observation carried forward (LOCF). Data not missing at random will be analyzed using mean substitution.

Both an intent-to-treat analysis and per protocol-analysis (PP analysis) will be performed

Revisions of SAP

None
